# Dr. Mary Elizabeth McCartney

**Published:** 1913-05

**Authors:** 


Dr. Mary Elizabeth McCartney, nee Edsall, formerly of Rochester, died April 11, at Avon Park, Fla.

